# The NADPH Oxidase Inhibitors Apocynin and Diphenyleneiodonium Protect Rats from LPS-Induced Pulmonary Inflammation

**DOI:** 10.3390/antiox12030770

**Published:** 2023-03-21

**Authors:** Ahmed Kouki, Wafa Ferjani, Néziha Ghanem-Boughanmi, Mossadok Ben-Attia, Pham My-Chan Dang, Abdelaziz Souli, Jamel El-Benna

**Affiliations:** 1 Centre de Recherche sur l’Inflammation, Laboratoire d’Excellence Inflamex, Faculté de Médecine Xavier Bichat, Université de Paris-Cité, INSERM-U1149, CNRS-ERL8252, F-75018 Paris, France; 2 Laboratoire de Biosurveillance de l’Environnement (LR01/ES14), Faculté des Sciences de Bizerte, Université de Carthage, Zarzouna 7021, Tunisia; 3 Unité des Risques Liés aux Stress Environnementaux (UR17/ES20), Faculté des Sciences de Bizerte, Université de Carthage, Zarzouna 7021, Tunisia

**Keywords:** NADPH-oxidase, apocynin, diphenyleneiodonium, DPI, LPS, lung inflammation, MPO, pro-inflammatory markers, oxidative stress

## Abstract

Inflammation is the body’s response to insults, for instance, lung inflammation is generally caused by pathogens or by exposure to pollutants, irritants and toxins. This process involves many inflammatory cells such as epithelial cells, monocytes, macrophages and neutrophils. These cells produce and release inflammatory mediators such as pro-inflammatory cytokines, lipids and reactive oxygen species (ROS). Lung epithelial cells and phagocytes (monocytes, macrophages and neutrophils) produce ROS mainly by the NADPH oxidase NOX1 and NOX2, respectively. The aim of this study was to investigate the effects of two NADPH oxidase inhibitors, apocynin and diphenyleneiodonium (DPI), on lipopolysaccharide (LPS)-induced lung inflammation in rats. Our results showed that apocynin and DPI attenuated the LPS-induced morphological and histological alterations of the lung, reduced edema and decreased lung permeability. The evaluation of oxidative stress markers in lung homogenates showed that apocynin and DPI inhibited LPS-induced NADPH oxidase activity, and restored superoxide dismutase (SOD) and catalase activity in the lung resulting in the reduction in LPS-induced protein and lipid oxidation. Additionally, apocynin and DPI decreased LPS-induced MPO activity in bronchoalveolar liquid and lung homogenates, TNF-α and IL-1β in rat plasma. NADPH oxidase inhibition could be a new therapeutic strategy for the treatment of inflammatory lung diseases.

## 1. Introduction

The lungs present the largest epithelial surface in direct contact with the outside environment. It is the primary target of many airborne pathogens, toxins and allergens that cause acute lung injury [1]. Inflammation of the lungs is generally caused by exposure to pathogens such as Gram-negative bacteria which release high quantities of lipopolysaccharide (LPS) [1,2]. This agent is the predominant driver of microbial inflammatory processes responsible for strong immune responses [3], leading to the production of reactive oxygen species (ROS) and pro-inflammatory cytokines, with increased infiltration of neutrophils [1,4]. Acute inflammation is characterized by the recruitment of neutrophils and massive lymphocytes into the interstitial tissue, rupture of the epithelial microarchitecture with parenchymal lesions and pulmonary edema [5]. The production of ROS is critical in the progression of inflammatory diseases. ROS act as signaling molecules and inflammatory mediators [6]. ROS are mainly generated from two main sources, the mitochondrial electron transport chain and the membrane enzymatic complexes, known as nicotinamide adenine dinucleotide phosphate (NADPH) oxidases (NOX). In the lungs, epithelial cells and phagocytes (monocytes, macrophages and neutrophils) produce ROS mainly by the NADPH oxidase NOX1 and NOX2, respectively [7]. NOX2 is involved in microbial killing and elimination by converting molecular oxygen to superoxide anion (O_2_^−^), which in turn is converted to antimicrobial ROS such as hydrogen peroxide (H_2_O_2_) and hypochlorous acid (HOCl) [8,9]. However, the dysregulation of NOX2 can lead to increased production of ROS contributing to acute or chronic lung inflammation [10,11]. The inhibition of NADPH oxidase could be useful to limit ROS production and inflammatory reaction.

**Figure 1:**
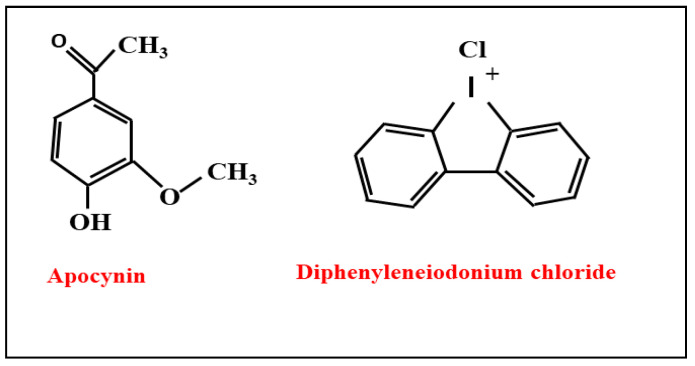
Chemical structures of apocynin and diphenyleneiodonium.

Several NADPH oxidase inhibitors were identified, the most known are apocynin and diphenyleneiodonium (DPI) [12,13] (Figure 1). Apocynin is a non-toxic phytochemical agent purified from the roots (rhizome) of the plant *Picrorhiza kurroa* (Scrofulariaceae) increasingly popular and inexpensive product due to its inhibitory effect on NADPH oxidase, and its antioxidant and anti-inflammatory properties [14,15]. In vitro and in vivo studies indicate that apocynin exerts anti-inflammatory effects through multiple molecular mechanisms [15]. Diphenyleneiodonium is a lipophilic reagent, that has high toxicity that limits its therapeutic applications to experimental diseases, and acts as an uncompetitive inhibitor of flavoenzymes, and as such NOXs [16,17,18,19]. DPI acts as a hypoglycemic agent that blocks glycogenesis and cellular respiration in the liver [20]. This inhibitor also acts at very low doses as a potent anti-inflammatory by reducing macrophagic-mediated inflammatory responses in the colorectal cancer model [21].

The main objective of this study is to test the effects of two NADPH oxidase inhibitors (Apocynin and DPI) on lung inflammation induced by intranasal administration of LPS in rats.

## 2. Materials and Methods

### 2.1. Reagents

Apocynin (4-hydroxy-3-methoxyacetophenone), Diphenyleneiodonium, DMSO (dimethyl sulfoxide), HTAB (hexadecyltrimethylammonium bromide), PBS (phosphate-buffered saline), LPS (Lipopolysaccharide from *E. coli* O111:B4), BSA (bovine serum albumin), Folin ciocalteu, chloroamine-T, Potassium iodide (KI), acetic acid, BHT (Butylated hydroxytoluene), TCA (trichloroacetic acid), TBA (thiobarbituric acid), HCL (Hydrochloric acid), bovine catalase, epinephrine, sodium carbonate/bicarbonate, orthodianisidine dihydrochloride, Tris buffer, Hydrogen peroxide, Luminol (5-amino2,3-dihydro-1,4-phtalazinedione), Horseradish peroxidase (HRP) and NADPH were from Sigma-Aldrich (St. Louis, MI, USA). Rat TNF-α ELISA Kit and Rat IL-1β ELISA Kit were from Peprotech (Cranbury, NJ, USA).

### 2.2. Animal Care

Male Wistar rat, weighing 213 ± 15 g, obtained from the “Pasteur Institute of Tunis’’. Animals were randomly divided into six groups of five animals; they were kept under controlled conditions throughout the experiments. Animals were fed standard rodent water ad libitum and kept under controlled temperature (22 ± 1 °C), humidity (65–70%), and a 12:12 h light-dark cycle throughout the experiments. All animal experiments were performed in compliance with the Tunisian code of practice for the care and use of animals for scientific purposes as well as the recommendations and the Council of Europe (1986) for the protection and use of vertebrate animals. The ethical committee of the Tunisian Association of Laboratory Animals Science (ATSAL) approved the experimental protocol (No. 0120/2022 ATSAL).

### 2.3. In Vivo Experimental Protocol

**Figure 2:**
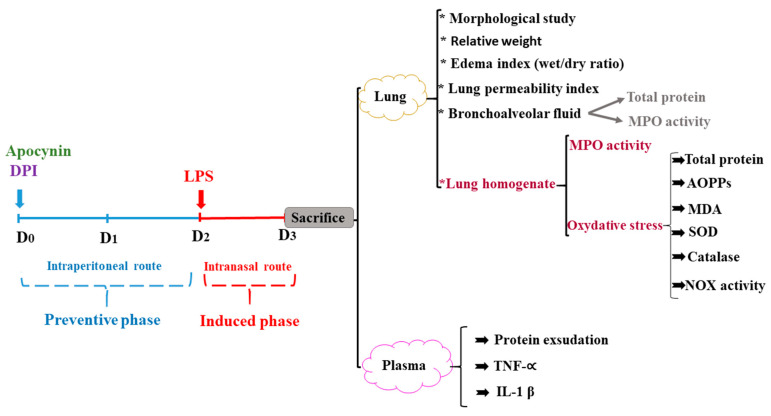
Schematic representation of the procedure for studying the effects of apocynin and DPI on acute lung inflammation induced by LPS.

The animals were divided into six groups of five rats, the two groups (control group and the LPS group) received a daily intraperitoneal (i.p.) injection of DMSO for two days with intranasal administration of PBS for the control group and the LPS group received intranasal administration of LPS (1.5 mg/mL). The AP and (AP + LPS) groups received a daily i.p. injection of Apocynin dissolved in DMSO (10 mg/kg) followed by intranasal administration of PBS for the AP group and LPS for (AP + LPS) group, and the last two groups DPI and (DPI + LPS) received a daily i.p. injection of DPI dissolved in DMSO (100 ng/kg) followed by intranasal administration of PBS for the DPI group and LPS for (DPI + LPS) group (Figure 2).

### 2.4. Body Weight Changes and Organ Measurement

In this study, we checked the body weight of the rats daily using an electronic scale. The rats’ water and food consumption levels were also controlled, as were the signs of stress such as the appearance of droppings and the decline in animal activity. At the end of this experiment, animals were sacrificed by decapitation. Plasma and organs were carefully collected their relative weights were measured as described above. The assessment of the edema degree was conducted by recording the relative weight of the lung of rats. This parameter was calculated according to El-Alfy et al., 2012 [22], using the following formula: “= [organ weight (g)/body weight (g)]”.

### 2.5. Bronchoalveolar Liquid (BAL) and Lung Permeability Index Determination

Both lungs were removed and ligated to allow the complete separation of the left and right lungs. They were cut along the trachea leading to the bifurcation of the trachea. Then, 300 µL of PBS were added to the left lung to fill all the lobes. The injected wash liquid was collected back into the syringe and washed twice. The BAL was centrifuged and transferred to the tubes and stored at 80 °C. The total protein concentration was measured. The lung permeability index was calculated as the ratio of the protein concentration in BAL and the protein concentration in plasma [23].

### 2.6. Lung Wet/Dry Weight Ratio

In order to evaluate the lung edema, the central lobe of the lung of each rat was removed and rinsed with 0.9% NaCl physiological solution and weighed. The lung tissue samples were then dried at 80 °C for 48 h to determine the dry weight and calculate the wet/dry ratio.

### 2.7. Histological Study

Lungs were fixed overnight in 10% formalin-PBS, dehydrated in graded ethanol solutions, and embedded in paraffin. Histological specimens were sectioned at 5 µm, stained with hematoxylin and eosin (H&E), and detected by light microscopy (Carl Zeiss, Jena, Germany).

### 2.8. Lung Homogenate and Protein Determination

Lung homogenate was rinsed in ice-cold NaCl 0.9 and homogenized in 0.1 M phosphate buffer (pH 7.4). The homogenates 10% (*w*/*v*) were centrifuged for 20 min at 4000× *g* rpm at 4 °C, and then supernatants were stored at −80 °C to be used for quantification of protein level, MDA content, SOD activity, catalase activity, AOPP level, myeloperoxidase activity, and NOX activity. Proteins were measured using the method of Lowry et al., 1951 [24].

### 2.9. NOX Activity

Lung homogenates are sonicated then a volume of 25 µL was resuspended in 202.5 μL of relaxation buffer and pre-incubated for 5 min at 37 °C in the presence of luminol (10 µM) and HRPO (500 U/mL). Samples were then stimulated with NADPH (3.2 mM) and luminol-enhanced chemiluminescence was measured by a luminometer (Biolumat LB937; Berthold, Bad Wildbad, Germany) for 10 min. The results were expressed as a % of the LPS group.

### 2.10. Advanced Oxidation of Protein

The evaluation of advanced oxidation of protein products (AOPP) is a spectrophotometric method described by Witko-Sarsat et al. (1984) [25]. AOPP level was evaluated based on a standard curve prepared with chloroamine T and potassium iodide (KI) and acetic acid and reading absorbance at 340 nm with a reference filter at 490 nm. AOPP concentration was expressed as µM/mg of protein equivalent according to the chloramines T standard curve.

### 2.11. Lipid Peroxidation Was Measured as Malondialdehyde

The lipid peroxidation was determined by MDA measurement according to Yagi, 1976 [26]. Briefly, aliquots of lung homogenates were mixed with BHT-trichloroacetic acid (TCA) solution containing 1% BHT (*w*/*v*) dissolved in 20% TCA (*w*/*v*) and centrifuged at 1000× *g* for 5 min at 4 °C. The supernatant was blended with a solution containing (0.5 N HCl, 120 mM TBA buffered in 26 mM Tris) and then heated at 80 °C for 10 min. After cooling, the absorbance was determined at 532 nm using a UV–visible spectrophotometer (Beckman DU 640B). MDA levels were expressed as nmol MDA · mg^−1^ protein (molar extinction coefficient: 1.56 × 10^5^ M^−1^ cm^−1^).

### 2.12. Superoxide Dismutase (SOD) and Catalase Activity

SOD activity was determined by using a modified epinephrine assay [27]. At alkaline pH, superoxide anion O_2_^−^ causes the autoxidation of epinephrine to adrenochrome while competing with this reaction, SOD decreased the adrenochrome formation. One unit of SOD is defined as the amount of the extract that inhibits the rate of adrenochrome formation by 50%. Enzyme extract was added in a 2 mL reaction mixture containing 10 μL of bovine catalase (0.4 μL), 20 μL epinephrine (5 mg/mL) and 62.5 mM sodium carbonate/bicarbonate buffer (0.1 M, pH 10.2). Changes in absorbance were recorded at 480 nm by a UV-visible spectrophotometer (Beckman DU 640B). Catalase activity [28] is based on the determination of the rate constant of hydrogen peroxide decomposition by the catalase enzyme. The decomposition of H_2_O_2_ was followed directly by monitoring the decrease in absorbance at 240 nm using a UV-visible spectrophotometer (Beckman DU 640B). Data were expressed as Mmol of H_2_O_2_ per mg^−1^ protein.

### 2.13. Assessment of Myeloperoxidase (MPO) Activity and Pro-Inflammatory Cytokines (IL-1β, TNF-α) Levels

Myeloperoxidase activity in BAL and the lung homogenate assay was determined according to this procedure; in brief tissue samples and BAL were mixed with HTAB (0.5%) were sonicated twice, frozen, and then centrifuged at 15,000× *g* for 15 min at 4 °C, and myeloperoxidase activity was assayed according to the method of Goldblum et al., 1985 [29]. After this procedure, 50 μL of each sample was mixed with phosphate buffer (350 μL) containing 0.167 mg/mL orthodianisidine dihydrochloride, incubated for 5 min and a 50 μL of hydrogen peroxide (0.0005%) was added for a total volume 500 μL. The change in absorbance at 460 nm was recorded by a spectrophotometer and was read every 30 s for 10 min. One unit (U) of MPO activity was defined as consuming 1 nmol of peroxide per minute at 22 °C. Data were expressed as % of MPO activity of the LPS group. Pro-inflammatory cytokine (IL-1β, TNF-α), levels were assayed in the plasma using ELISA kits, according to the manufacturer’s instructions.

### 2.14. Statistical Analysis

All results are expressed as mean ± standard error (S.E.M.). Data were analyzed using Graph-Pad Prism 7 software (Graph-Pad Software, San Diego, CA, USA). Differences between groups were analyzed by one-way analysis of variance (ANOVA) followed by Tukey’s multiple comparison post-test. * *p* < 0.05, ** *p* < 0.01 and *** *p* < 0.001 values were considered significant.

## 3. Results

### 3.1. Apocynin and DPI, at 10 mg/kg and 100 ng/kg Respectively, Are Not Toxic for Rats

In order to assess the effects of NOXs inhibitors on lung inflammation in rats, animals were not treated or treated with apocynin (10 mg/kg) or DPI (100 ng/kg) for two days, then subjected to intranasal administration of LPS for one day to induce acute lung inflammation, then were sacrificed, and different parameters were assessed following the experimental diagram shown in Figure 2. The concentrations of apocynin and DPI used in vivo in this study have been tested in rats without any toxic effect [30].

The determination of the rats’ body weight showed (Table 1), a slight variation in the body weight in each group during the first two days of the study. This variation is not significant even after the administration of LPS. For the DPI + LPS group, our results show that the pretreatment by DPI induced a significant reduction (*p* < 0.05) in the mean body weight as compared to day 1; however, treatment with LPS did not further induce body weight loss. These results indicated that the treatment of rats with apocynin and DPI then LPS was well tolerated.

### 3.2. Morphological and Histological Evaluation of the Effects of Apocynin and DPI on Rat Lung in the Absence or Presence of LPS

**Figure 3:**
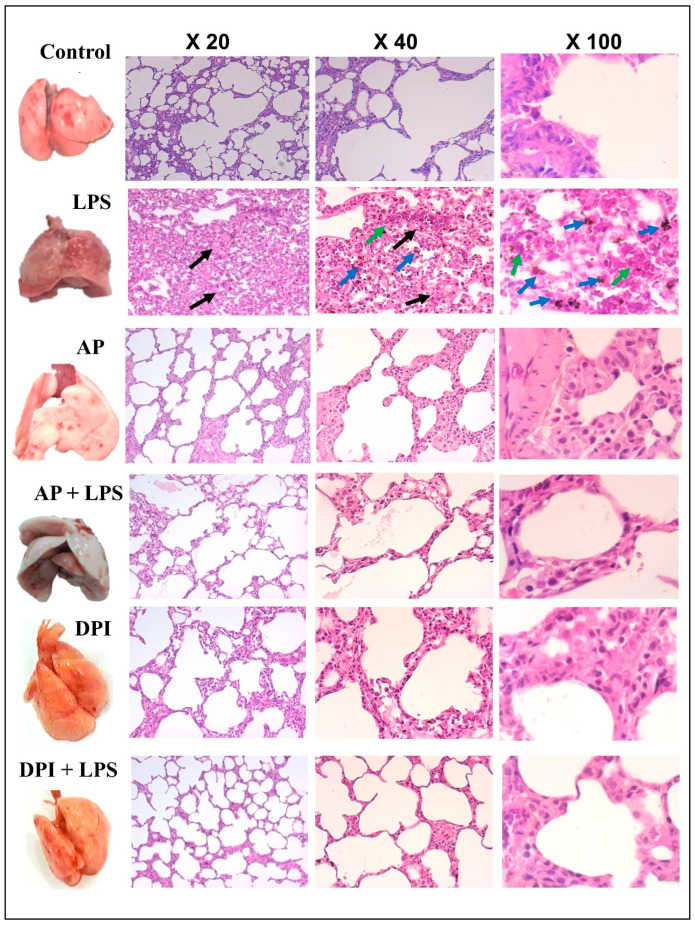
Morphological and histological evaluation of the lungs in different groups: Images of different magnifications are displayed. Blue: erythrocyte aggregation; green: intense neutrophil; black: pulmonary edema. For each condition image of one rat lung is representative of five rats.

After rat sacrifice, the morphology of the lungs was first evaluated (Figure 3, Left line). Results show that LPS induced a redness of the lung, a sign of inflammation. Interestingly, i.p. administration of apocynin or DPI before LPS treatment prevented this redness. Second, histology analysis of the lungs after hematoxylin and eosin staining at different magnifications indicates that LPS induced pulmonary edema (Figure 3). This edema is marked by thickened alveolar walls (black arrows, magnification X20 and X40) and alveolar infiltrate rich with neutrophils (green arrows), which leads to an alteration in tissue microarchitecture (magnification X40). Additionally, intranasal administration of LPS induced lung inflammation manifested by the aggregation of erythrocytes with intra-alveolar bleeding (blue arrows, magnification X40 and X100). Apocynin or DPI alone did not affect lung tissue structure and did not result in any accumulation of inflammatory cells. The pre-treatments by apocynin or DPI prevented the accumulation of inflammatory cells in alveolar infiltrates with the attenuation of the pulmonary edema caused by the LPS (Figure 3).

**Figure 4:**
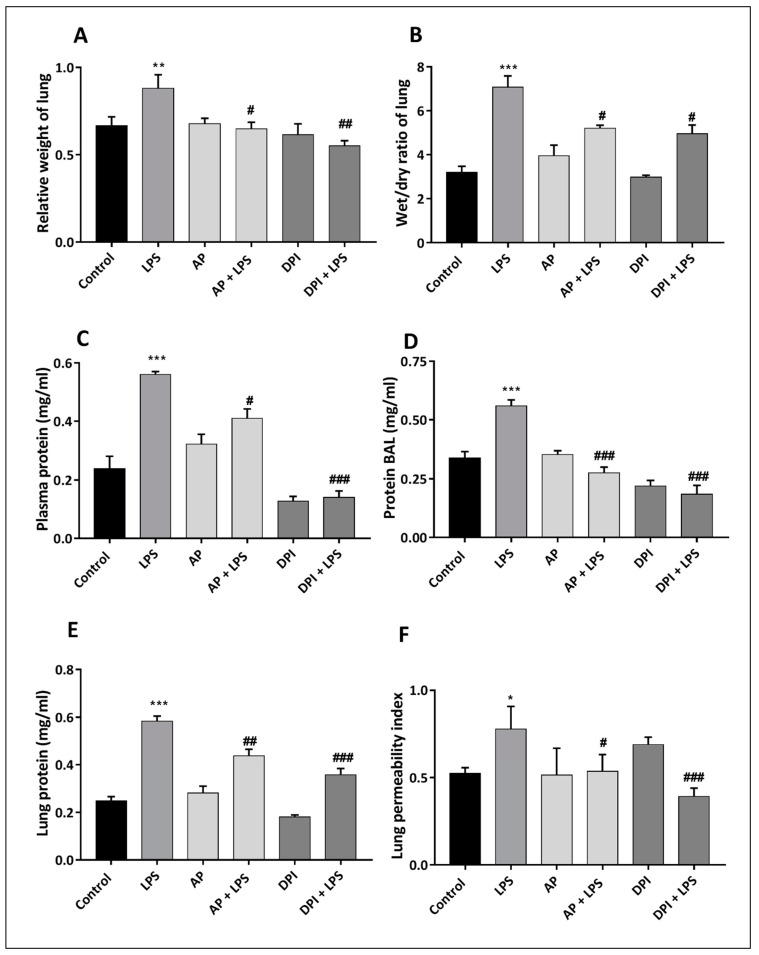
Effect of apocynin (AP) and DPI on relative weight of lung (**A**), wet/dry ratio of lung (**B**), total protein in plasma (**C**), proteins in BAL (**D**), lung protein (**E**) and lung permeability index (**F**) during the induction of acute lung inflammation by LPS, the results are presented in the form of means ± S.E.M with *n* = 5 with * *p* < 0.05, ** *p* < 0.01 and *** *p* < 0.001 compared to the control group and **^#^** *p* < 0.05, **^##^** *p* < 0,01 and ^###^ *p* < 0.001 compared to the LPS group.

Quantitative analysis showed that the intranasal administration of LPS causes a significant increase in the relative weight of the lungs (as calculated by the Ratio: lung weight/Rat body weight) compared to the control group, while the pre-treatment with apocynin alone or DPI alone did not affect the relative lung weights compared to the control group. Our results indicate that the pre-treatment with apocynin followed by intranasal administration of LPS and (AP + LPS) leads to a significant decrease in this parameter compared to the LPS group, also for (DPI + LPS) group results showed a significant decrease (*p* < 0.01) in the relative weight of lung compared to LPS group (Figure 4A).

The evaluation of pulmonary edema index by the determination of the wet/dry weight ratio of the lung showed that the LPS increased significantly this ratio compared to the control group. Additionally, our analysis indicates that the i.p. administration of apocynin (AP group) or DPI (DPI group) does not affect this parameter compared to the control group (*p* > 0.12) Our results showed that the pre-treatment with apocynin (AP + LPS group) and DPI (DPI + LPS group) showed significant protection against pulmonary edema induced by LPS (*p* < 0.01) (Figure 4B).

The assessment of total protein levels in plasma shows that LPS leads to a significant increase (*p* < 0.001) in total plasmatic protein level (0.560 ± 0.01 mg/mL) compared to the control group (0.238 ± 0.04 mg/mL). The i.p. administration of apocynin alone showed a weak but not significant increase in the level of total proteins (0.323 ± 0.032 mg/mL) compared with the control group. However, DPI significantly reduced (*p* < 0.001) the level of total proteins (0.091 ± 0.007 mg/mL) compared to the control group. Therefore, the pretreatment with apocynin followed by intranasal administration of LPS induced a significant decrease (*p* < 0.05) in total plasma proteins caused by LPS. Additionally, our results showed that DPI significantly decreased (*p* < 0.001) the level of total protein in the plasma compared to the LPS group (Figure 4C).

The evaluation of total proteins in the bronchoalveolar lavage (BAL) fluid showed that intranasal administration of LPS causes a significant increase (*p* < 0.001) in total BAL proteins level (0.560 ± 0.025 mg/mL) in comparison to the control group (0.339 ± 0.026 mg/mL). The i.p. administration of apocynin (AP group) or DPI (DPI group) did not affect total protein compared to the control group (*p* > 0.5). Pretreatment with apocynin followed by intranasal administration of LPS (AP + LPS) leads to a significant decrease (*p* < 0.001) in total proteins in the BAL (0.275 ± 0.025 mg/mL) compared to LPS. Additionally, our results indicate that DPI protected against the increase in the level of total BAL proteins caused by LPS (Figure 4D).

The determination of tissue protein levels in lung homogenate showed that LPS causes a significant increase (*p* < 0.001) in tissue protein levels compared to the control group. Additionally, apocynin alone (AP group) or the treatment with DPI without LPS (DPI group) does not result in any change in protein content compared to the control group (*p* > 0.1). The pre-treatment by apocynin (AP + DPI) results in a significant reduction (*p* < 0.01) in protein level compared to the LPS group. Our results showed also that the pre-treatment by the DPI (DPI + LPS) results in a significant reduction (*p* < 0.001) in protein level, compared to the LPS group (Figure 4E).

LPS increased significantly (*p* < 0.05) the lung permeability index (0.777 ± 0.064) compared to the control group (0.523 ± 0.033). The treatment with apocynin alone (AP group) did not affect the level of this parameter (0.513 ± 0.068) compared to the control group, DPI alone had less effect (0.689 ± 0.024) but was not significant on the lung permeability index compared to the control group. The pretreatment with apocynin (AP + LPS) and DPI (DPI + LPS) significantly reduced the increase in the lung permeability index caused by LPS (Figure 4F).

### 3.3. Apocynin and DPI Reduce LPS-Induced Oxidative Stress in Rat

**Figure 5:**
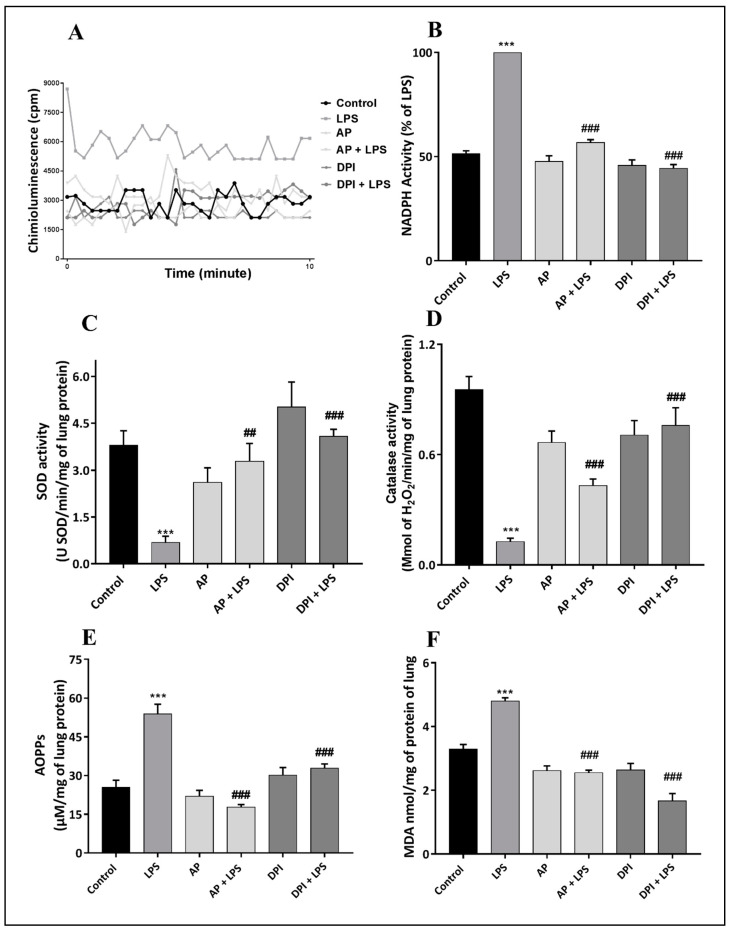
Effect of AP and DPI on NOX activity in lung homogenate (**A**) with data are expressed as mean ± S.E.M (**B**), SOD (**C**), catalase activity (**D**), AOPPs level (**E**), MDA (**F**) during the induction of acute lung inflammation by LPS. Data are displayed as mean ± SEM and represent at least five (*n* = 5) independent experiments. *** *p* < 0.001 significant differences as compared to the control group, **^##^** *p* < 0.001 and ^###^ *p* < 0.001 significant differences as compared to the LPS group.

We next wanted to check whether LPS induced oxidative stress in the lung and to assess the effect of apocynin and DPI on this process. First, we measured NADPH oxidase activation in lung homogenates. Results show that LPS increased NADPH oxidase activity in the lung and the i.p. administration of apocynin or DPI inhibited NADPH oxidase-derived ROS production following the administration of LPS (Figure 5A,B). Second, we measured the activity of SOD and catalase, two major ROS-catabolizing enzymes. The determination of superoxide dismutase in lung homogenates indicates that the treatment with LPS causes a significant decrease in this parameter compared with the control group (Figure 5C). The i.p. administration of apocynin alone (AP group) or DPI alone (DPI group) did not induce any variation in SOD activity compared to the control group. However, our results show that apocynin or DPI administration to rats, followed by an intranasal administration of LPS significantly prevented the decrease in SOD activity triggered by LPS (Figure 5C). The determination of catalase activity in the lung homogenate indicates that LPS causes a significant decrease in this activity (*p* < 0.001) compared to the control group (Figure 5D). Additionally, our results report that apocynin and DPI do not affect the activity of catalase compared to the control group. Apocynin and DPI significantly reduced (*p* < 0.001) the LPS-induced decrease in the catalase activity (Figure 5D). Additionally, our results report that apocynin and DPI do not affect the activity of catalase compared to the control group. While apocynin and DPI significantly reduced (*p* < 0.001) the LPS-induced decrease in the catalase activity (Figure 5D). These results indicate that LPS can induce an imbalance in ROS production and ROS catabolism resulting in excessive ROS accumulation and oxidative stress in the lung and that apocynin and DPI inhibit this process.

Oxidative stress can induce protein and lipid oxidation. The evaluation of the advanced oxidation status of proteins in lung tissue homogenate showed that intranasal LPS causes a significant increase (*p* < 0.001) in protein oxidation level compared to the control group while the i.p. administration of apocynin or DPI alone does not result in any change in the oxidation status of proteins. However, our results indicate that apocynin and DPI protected against the increase of the advanced oxidation of proteins induced by LPS (Figure 5E).

The measurement of the level of lipid peroxidation (MDA) in lung tissue indicates that LPS caused a significant increase (*p* < 0.001) in MDA content compared with the control group; i.p. administration of apocynin (AP group) or DPI (DPI group) did not alter the marker of lipid peroxidation compared to the control group. The DPI group decreased (*p* < 0.001) the level of this peroxidation significantly compared to the control group. Administration by the i.p. route of these two NADPH inhibitors followed by intranasal administration of LPS showed that both inhibitors protected rats against the increase in lipid peroxidation caused by LPS (Figure 5F).

### 3.4. Apocynin and DPI Reduce LPS-Induced Increase in Mediators of Inflammation Levels in Rat

**Figure 6:**
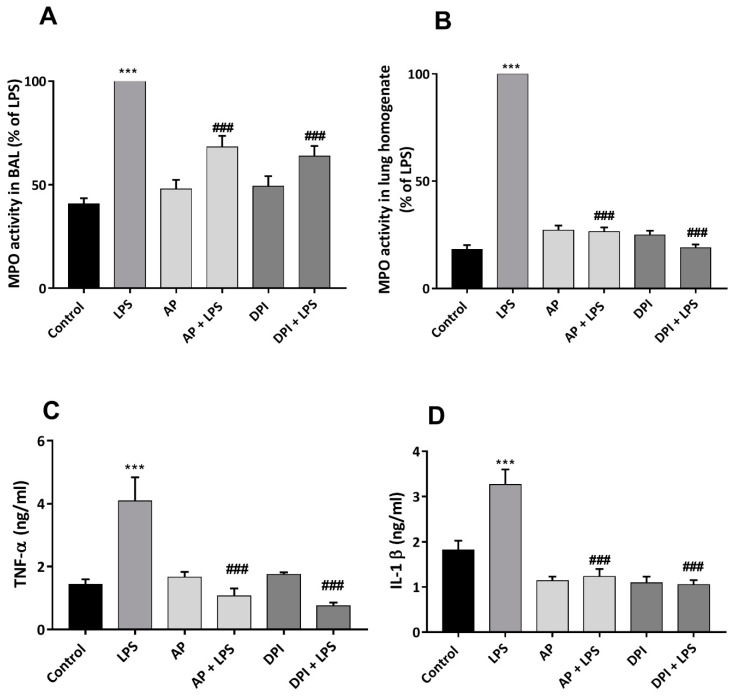
Effect of apocynin and DPI on the inflammatory markers MPO activity in BAL (**A**), lung homogenate (**B**), TNF-α (**C**) and IL-1β (**D**) during the induction of acute lung inflammation by LPS. Data are displayed as mean ± SEM and represent at least five (*n* = 5) independent experiments. *** *p* < 0.001 significant differences as compared to the control group ^###^ *p* < 0.001 significant differences as compared to the LPS group.

Lung inflammation is characterized by neutrophils in the lungs, a major inflammatory parameter. Myeloperoxidase (MPO) is a specific quantifiable marker of neutrophils. To quantify this inflammatory parameter, we determined MPO activity in BAL. Results showed that LPS induced a significant increase (*p* < 0.001) in MPO activity compared to the control group. Apocynin alone or the DPI did not affect the MPO activity compared to the control group. However, our results showed that both apocynin and DPI prevented the LPS-induced increase in MPO activities in BAL compared to the LPS group (Figure 6A). This result was further confirmed by the assessment of MPO activity in lung homogenates, indicating that intranasal administration of LPS results in a significant increase of 81.84% in myeloperoxidase activity relative to the control group. The pretreatment with apocynin (AP group) and DPI (DPI group) did not affect the level of MPO activity compared to the control group. However, apocynin and DPI significantly attenuated the increase in myeloperoxidase activity by LPS (Figure 6B).

The determination of pro-inflammatory cytokines in rat plasma indicates that LPS triggers a significant increase (*p* < 0.001) in TNF-α level (4.09 ± 0.753 ng/mL) compared to the control group (1.432 ± 1.62 ng/mL) (Figure 6C). The i.p. administration of apocynin or DPI alone did not cause significant changes in the TNF-α level compared with the control group. On the other hand, apocynin (AP + LPS) and DPI (DPI + LPS) significantly reduced (*p* < 0.001) TNF-α levels after intranasal exposure to LPS compared to the LPS group (Figure 6C)**.**

An evaluation of interleukin (IL-1β) levels in rat plasma shows that LPS induces a significant increase (*p* < 0.001) in this pro-inflammatory marker compared with the control group (Figure 6D). The pre-treatment with apocynin triggers a significant reduction (*p* < 0.05) of 75.75% at the plasma level of IL-1ß compared with the control group. The i.p. administration of apocynin does not change the level of this marker compared to the control group. For the group (AP + LPS) our results showed that pretreatment with this inhibitor decreased the level of IL-1β by 66.75% compared to the LPS group. Thus, DPI significantly protects against the increase in IL-1β levels caused by LPS; this inhibitor reduces the secretion of interleukin IL-1β by 68% compared to the LPS group (Figure 6D).

## 4. Discussion

Excessive ROS production can induce tissue injury implicated in inflammatory diseases such as lung inflammatory diseases, inflammatory bowel diseases and inflammatory articular diseases. The family enzymes NADPH oxidases (NOXs) are major ROS-generating enzymes in several cells such as phagocytes, epithelial cells, endothelial cells and neurons. Here, we evaluated the effect of two known NOX inhibitors, apocynin and DPI on LPS-induced lung inflammation in rats. We found that apocynin and DPI protected rats from LPS-induced lung inflammation, inhibited ROS production and oxidative stress in the lungs and inhibited the LPS-mediated increase in the levels of the inflammatory mediators in rats.

The evaluation of the relative weight of the lungs during the induction of acute pulmonary inflammation by LPS shows that the intranasal administration of LPS triggers a significant increase in the relative weight of the lung compared with the control group. Additionally, the determination of the wet/dry ratio of the lung as well as the index of the lung permeability shows that the LPS causes pulmonary edema with an alteration of the lung permeability. Our results are in agreement with the study of Kumari et al., 2015, which showed that intranasal administration of lipopolysaccharide causes severe neutrophilia, alveolar protein extravasation and tissue damage, leading to pulmonary dysfunction [31]. The results of this study of total protein profile at different cell compartments such as lung homogenate, plasma level and also at the level of the bronchoalveolar fluid showed that LPS triggers an important protein influx during its administration which is a sign of acute pulmonary inflammation. Our results are consistent with the results of Ding Q et al., 2017 [32] which showed that LPS causes a significant increase in the protein level of bronchoalveolar fluid in LPS-treated mice. Interestingly, apocynin and DPI protected rats from lung tissue alteration and lung edema.

In addition, acute pulmonary inflammation induced by LPS leads to an imbalance in oxidation and reduction status which leads to damage to DNA and proteins with intense lipid oxidation [33]. The assessment of the oxidative status in lung homogenates shows that LPS causes an imbalance in oxidative status characterized by a significant increase in NADPH oxidase activity, and a decrease in SOD and catalase activity resulting in the increase in certain parameters of oxidative stress such as protein oxidation (AOPP) and lipid peroxidation (MDA) in the lungs. Interestingly, apocynin and DPI inhibited this LPS-induced oxidative stress in the lung. Several cells expressing NADPH oxidases can be present in the lungs, such as macrophages, neutrophils, dendritic cells and epithelial cells [34]. Macrophages, neutrophils, and dendritic cells express mainly NOX2 and epithelial cells express NOX1, both can be inhibited by apocynin and DPI [35].

Pro-inflammatory cytokines play a key role in inducing pulmonary inflammation [36]. In our study, we showed that LPS-induced pulmonary inflammation causes a significant increase in pro-inflammatory markers such as myeloperoxidase (MPO) from neutrophils, TNF-α and IL-1β. Our results are supported by the literature which shows that LPS causes an increase in pro-inflammatory cytokines such as TNF-α, IL-1β and IL-6 [37]. Indeed, TNF-α is a major pro-inflammatory cytokine leading to the amplification of the inflammatory response [38] while interleukin 1-ß causes the alteration of the epithelial layer with a direct action on the endothelial cells [39]. Apocynin and DPI inhibited the increase in the levels of these markers suggesting that NOX-derived ROS control TNF-α and IL-1β production in LPS-induced inflammation in rats.

Our results also show that DPI and apocynin exert protective effects against the production of ROS by the NADPH oxidase system in the lungs. These inhibitors inhibit NADPH oxidase through different mechanisms. Indeed, DPI is a flavoprotein inhibitor that can inhibit all the NOXs and even other flavoproteins, while apocynin can prevent the translocation of p47phox to the membranes and the assembly of the NOX2 complex [35]. The inhibitory effect of apocynin requires the presence of MPO in the cell or in the medium [40]. It was also reported that apocynin can react directly with ROS, thus inhibiting extracellular ROS propagation and inhibiting endothelial lesions becomes a powerful anti-inflammatory agent widely used in many diseases for curative or preventive purposes [15]. For example, in lung lesions, this molecule acts in a protective way against vascular permeability in the lungs [41,42]. Our results show that apocynin significantly inhibits the level of key pro-inflammatory markers such as TNF-α, interleukin 1-β and myeloperoxidase activity. It was shown that apocynin prevents neutrophil penetration into the lungs and inhibits the production of pro-inflammatory factors [43].

Additionally, apocynin is a natural phytochemical, non-toxic extract used at the milligram scale in the treatment of various pathologies [14]. On the other hand, ultra-low doses of DPI on a nanogram scale have protective effects and are used for the treatment of colitis [21]. Our results indicate that DPI showed a protective effect against endotoxin-induced lung inflammation. This protection is demonstrated by a reduction in lung permeability and LPS-induced edema.

## 5. Conclusions

In the present study, we show that intranasal administration of LPS for 24 h initiates acute pulmonary inflammation as shown by morphological and histological alterations as well as an increase in inflammatory markers such as oxidative stress, neutrophilia and pro-inflammatory cytokines. The use of NADPH oxidase inhibitors, apocynin and DPI protected rats from acute pulmonary inflammation and normalized inflammatory markers in rats. Inhibitors of NADPH oxidase could be useful new therapeutic agents for lung inflammatory diseases.

## Figures and Tables

**Table 1:** Regular assessment of rat body weight (g) during pretreatment with apocynin, DPI and intranasal administration of LPS. Results are presented as mean ± S.E.M. (*n* = 5 independent replicates). * *p* < 0.05, compared to the control group.

	Group	Control	LPS	AP	AP + LPS	DPI	DPI + LPS
Phase							
Preventive phase	Day 1	215.05 ± 5.7	226.3 ± 6.7	196.71 ± 2.4	206.45 ± 8.9	232.53 ± 7.5	198.73 ± 1.6
	Day 2	208.25 ± 6.1	223.19 ± 5.1	196.05 ± 1.9	203.3 ± 9.33	220.13 ± 6.2	189.95 ± 2.05 *
Induced phase	Day 3	202.33 ± 5.8	218.18 ± 3.9	191.44 ± 2.3	194.88 ± 8.9	212.98 ± 5.7	187.93 ± 2.07

## Data Availability

All data presented in this research are available in the article. Other inquiries can be directed to the corresponding authors.

## Abbreviations

AOPP	Advanced oxidation protein products
AP	Apocynin
ATSAL	Tunisian Association of Laboratory Animals Science
BAL	Broncho alveolar fluid
BHT	Butylated hydroxytoluene
BSA	Bovine serum albumin
DMSO	Dimethyl sulfoxide
DPI	Diphenyleneiodonium
HRP	Horseradish peroxidase
HTAB	Hexadecyltrimethylammonuim bromide
i.p.	intraperitoneal
KI	Potassium iodide
LPS	Lipopolysaccharide
Luminol	(5-amino2,3-dihydro-1,4-phtalazinedione)
MDA	Malondialdehyde
MPO	Myeloperoxidase activity
NADPH	Nicotinamide adenine dinucleotide phosphate
NOXs	NADPH oxidases
PBS	Phosphate-buffered saline
ROS	Reactive Oxygen Species
SOD	Superoxide dismutase
TBA	Thiobarbituric acid
TCA	Trichloroacetic acid
